# Relevance of Cardiovascular Exercise in Cancer and Cancer Therapy-Related Cardiac Dysfunction

**DOI:** 10.1007/s11897-024-00662-0

**Published:** 2024-05-02

**Authors:** Simon Wernhart, Tienush Rassaf

**Affiliations:** Department of Cardiology and Vascular Medicine, West German Heart- and Vascular Center, University Hospital Essen, University Duisburg-Essen, Hufelandstrasse 55, 45147 Essen, Germany

**Keywords:** Cardiorespiratory fitness, Cardiotoxicity, Heart failure, Exercise

## Abstract

**Purpose of the Review:**

Cancer therapy-related cardiac dysfunction (CTRCD) has been identified as a threat to overall and cancer-related survival. Although aerobic exercise training (AET) has been shown to improve cardiorespiratory fitness (CRF), the relationship between specific exercise regimens and cancer survival, heart failure development, and reduction of CTRCD is unclear. In this review, we discuss the impact of AET on molecular pathways and the current literature of sports in the field of cardio-oncology.

**Recent Findings:**

Cardio-oncological exercise trials have focused on variations of AET intensity by using moderate continuous and high intensity interval training, which are applicable, safe, and effective approaches to improve CRF.

**Summary:**

AET increases CRF, reduces cardiovascular morbidity and heart failure hospitalization and should thus be implemented as an adjunct to standard cancer therapy, although its long-term effect on CTRCD remains unknown. Despite modulating diverse molecular pathways, it remains unknown which exercise regimen, including variations of AET duration and frequency, is most suited to facilitate peripheral and central adaptations to exercise and improve survival in cancer patients.

## Introduction

Cancer poses a major health and economic burden worldwide and warrants further improvement of diagnostic and treatment measures [1, 2]. Prognosis of cancer patients depends not only on adequate and timely diagnostics and treatment of cancer but also on handling of cardiovascular co-morbidities [3, 4]. Improvement or stabilizing of cardiorespiratory fitness (CRF), which is an independent predictor of cardiovascular events and mortality [5], has attracted much attention in recent years as a potential tool to interfere with cancer survival, cancer recurrence, as well as prevention and treatment of cardiovascular diseases associated with cancer and cancer therapy [6, 7]. A growing number of home-based and supervised exercise interventions, ranging from resistance training (RT) to aerobic exercise training (AET) in the form of either moderate continuous (MCT) or high intensity interval training (HIIT), have become available in clinical medicine and cardio-oncology. In this review we discuss the current literature on sports in cardio-oncology and the impact of AET on cardiovascular morbidity, heart failure hospitalization, and cancer therapy-related cardiac dysfunction (CTRCD). We further discuss molecular mechanisms associated with exercise-induced modification of cellular pathways. We comment on potential adaptations of training regimens to improve central and peripheral adaptations of exercise in cancer patients.

## Definition of Cancer Therapy-Related Cardiac Dysfunction

CTRCD is a descriptive term incorporating potential presentations of cancer patients including cardiac injury, heart failure, immune checkpoint inhibitor-related myocarditis, or cardiomyopathy [8]. CTRCD can be either asymptomatic or display symptoms of different severity (mild to very severe) [8]. Symptomatic CTRCD contains heart failure (HF) as a clinical syndrome consisting of cardinal clinical symptoms (e.g., breathlessness or fatigue) that may be accompanied by clinical signs of HF (e.g., peripheral oedema) and is divided into categories based on the measurement of left ventricular ejection fraction, LVEF (heart failure with reduced ejection fraction, HFrEF, LVEF ≤ 40%; heart failure with mildly reduced ejection fraction, HFmrEF, LVEF 41–49%; heart failure with preserved ejection fraction, HFpEF, LVEF ≥ 50%) [8–10]. Asymptomatic CTRCD requires an LVEF ≥ 50% and a new relative decline in global longitudinal strain (GLS) by > 15% from baseline and/or a new rise in cardiac biomarkers (defined as cardiac Troponin I or T above the 99th percentile, a BNP ≥ 35 pg/mL, an NT-proBNP ≥ 125 pg/mL, or a new significant rise from baseline beyond the biological and analytical variation of the assay used) [8]. Moderate, asymptomatic CTRCD refers to a new LVEF reduction by ≥ 10 percentage points to an LVEF of 40–49%, or a new LVEF reduction by < 10 percentage points to an LVEF of 40– 49% and either a new relative decline in GLS by > 15% from baseline, or a new rise in cardiac biomarkers, as defined above. Severe asymptomatic CTRCD is diagnosed if a new LVEF reduction to < 40% is observed [8].

Patients undergoing anthracycline-based (AC-based) chemotherapy and showing elevated cardiac troponin levels are more prone to developing CTRCD [11]. Likewise, a study on HER-2 positive breast cancer patients receiving trastuzumab demonstrated that 19% of patients who developed CTRCD during treatment displayed positive troponin levels at baseline, and troponin was a predictor of a lack of recovery despite optimized heart failure treatment [12]. Repetitive measurements of troponin during trastuzumab therapy revealed that increased baseline troponin was associated with a fourfold risk of left ventricular dysfunction [13]. However, it needs to be considered that baseline troponin in these studies represents pre-trastuzumab and post-AC therapy states. Thus, the impact of elevated baseline troponin levels in therapy-naïve breast cancer patients (before initiation of AC-based therapy) on the development of left ventricular dysfunction needs to be studied more deeply.

## Cardiovascular Morbidity and Heart Failure in Cancer

A diagnosis of cancer has been independently associated with an enhanced risk of cardiovascular mortality, stroke, HF, and pulmonary embolism [14•, 15–18]. After a follow-up of more than 25 years breast cancer survivors of the CLUE II cohort displayed a significantly higher risk of death from cardiovascular disease compared to a cancer-free control group (HR = 1.65, CI = 1.00 to 2.73) [19]. On top of chemotherapy-induced cardiotoxicity, radiation therapy also predisposes to a higher prevalence of cardiovascular events [20].

Decline of LVEF often occurs within the first year of cancer treatment and has been acknowledged as a threat to long-term morbidity in patients undergoing cardiotoxic (e.g., AC-based) chemotherapy with or without overt signs of HF [8, 21–23]. This has led to early screening and surveillance protocols (for instance using the HFA-ICOS risk assessment tool) to identify high-risk patients [22, 24]. These protocols, derived from the definition of CTRCD, implement echocardiographic variables, such as GLS [25] and LVEF, as well as biomarkers, such as NTproBNP and troponin [8, 26–28]. Higher troponin levels have been associated with a fourfold risk of developing left ventricular (LV) dysfunction in breast cancer (BC) patients following AC-therapy [13]. There is conflicting data on the role of baseline troponin and NTproBNP on all-cause mortality across tumor entities [29, 30]. Variables of resting echocardiography have also demonstrated prognostic value in cancer patients. Increased baseline indexed left ventricular end-diastolic volume was shown to be a predictor of major cardiovascular events, defined as symptomatic HF or cardiac death, during AC-based chemotherapy in patients with preserved LVEF [31]. Baseline GLS can predict left ventricular dysfunction in patients receiving AC and/or trastuzumab [32, 33].

In breast cancer survivors (CS) the clinical incidence of HF has been estimated to be around 2%, while a subclinicial cardiac dysfunction following AC-based treatment was observed in 10% [34]. Extensive reviews demonstrated an incidence of HF of 5% if AC therapy was applied > 400 mg/m^2^ [35]. Early introduction of HF medication and meticulous follow-up in patients showing cardiotoxicity has shown potential to improve LVEF [23, 36, 37], although decreased LVEF, pulmonary hypertension, enlarged left ventricular size, and anemia were shown to be negative long-term prognosticators [38]. If untreated, early-onset, progressive AC-based cardiotoxicity was shown to have comparable cardiovascular mortality to idiopathic dilated cardiomyopathy (IDCM) with an LVEF < 50% (overall survival rates at 5 and 10 years: cancer patients 86% and 61% and IDCM 88% and 75%; *p* = 0.61) [39] and was demonstrated to be dose-dependent [40].

## Impact of Cardiorespiratory Fitness on Cancer

Higher cardiorespiratory fitness (CRF), which is usually expressed through peak oxygen consumption (VO_2peak_), has been associated with lower cancer incidence [41] and better survival in large cohorts of cancer patients [6, 42]. The risk of cardiovascular disease-related mortality in CS is reduced by 14% per 1 metabolic equivalent (3.5 ml O_2_/kg/min) increase in CRF [43•]. In a single-center study of adult-onset cancer patients with a median follow-up of almost five years, higher CRF was an independent predictor of overall, cancer-related, and cardiovascular mortality [43•]. Higher (self-reported) CRF prior to cancer diagnosis was associated with a graded reduction on cardiovascular events in a large prospective study with long-term breast CS [44]. Late cardiac effects of AC-based therapy were prospectively investigated in female breast cancer patients without cardiovascular risk factors: Physically active individuals reported less symptoms of HF compared to sedentary controls after 5 years [45].

The ability of exercise intervention to prevent CTRCD, as it is defined currently, is less clear. In a meta-analysis, eight studies were analyzed in which LVEF and GLS were measured prior and after chemotherapy in BC patients [46]. GLS and LVEF did not differ between groups with or without exercise. CTRCD incidence was only reported in two out of eight trials [47, 48], with two cumulative patients in the UC groups diagnosed with CTRCD. It has been suggested that the reason for the lack of CTRCD prevention through exercise are short follow-ups, the heterogeneity of applied exercise protocols, some of which were not based on cardiopulmonary exercise testing (CPET), and the failure to adequately phenotype patients [49, 50•]. Exercise prescriptions were largely generic and based on predicted maximal heart rate, which may be insufficient to characterize adequate training corridors in patients on [51] and off [52] drug therapy.

## Exercise Interventions in Cancer Patients

The gold standard to determine training corridors for structured, individualized exercise training in healthy individuals is CPET [53–55]. In oncology, CPET has primarily been used to conduct pre-operative risk stratification in colon [56], rectal [57], and lung cancer [58], but not as a standard to design training intensity for exercise trials. Recommendations for exercise prescriptions in cancer patients have already been published using the subjective BORG scale [59], percentage of VO_2peak_, percentage of peak heart rate, or blood lactate levels as approaches to determine training corridors for exercise intensity [49, 60, 61]. Although formulae to predict peak heart rate [62] and VO_2peak_ [63] have been suggested, individual exercise testing prior to training is the most accurate approach and mandatory to improve (the lack of) comparability of trials in sports cardio-oncology. Apart from VO_2peak_, which is exertion-dependent, application of submaximal CPET variables, such as the oxygen equivalent at the first ventilatory threshold (VT1), or percentage of VO_2peak_ at VT1, may bear the potential to prescribe exercise intensities without the need for maximal exertion and may reduce adverse events during strenuous exercise [55, 64, 65•]. This, however, needs to be further studied in a cancer population.

The methodological heterogeneity of exercise trials in sports cardio-oncology is demonstrated by one of the first retrospective, exploratory trials (a sub-analysis of the HF-ACTION trial with LVEF ≤ 35%) to investigate AET (three supervised sessions/week, 60–70% of heart rate reserve, HRR, for 20–45 min) in cancer, which found cardiovascular mortality or HF hospitalization to be higher in AET after a median follow-up of 35 months (41 vs 67%, adjusted HR 1.94, CI 1.12–3.16, *p* = 0.017) [66], while VO_2peak_ did not differ compared to usual care (UC, *p* = 0.710). This intention-to-treat analysis was not powered for the primary endpoint and information on anticancer therapy, cancer stage, and type were not collected. These (hypothesis-generating) results were refuted by several trials showing a beneficial effect of physical activity (PA = unstructured activity vs. structured AET) on mortality: A meta-analysis of 16 exercise trials on breast and colon cancer survivors demonstrated a reduced mortality rate if patients increased their PA from pre-to post-diagnosis (RR = 0.61; CI = 0.46–0.80).

There is a large body of data supporting an increase of CRF through AET in randomized controlled trials in cancer patients after chemotherapy [67, 68], while mitigation of chemotherapy-induced reduction of VO_2peak_ has been demonstrated in high-volume AET as opposed to UC (2.5 ml/kg/min vs. 3.4 ml/kg/min) without significant side effects [69], and may even lead to an increase of VO_2peak_ during chemotherapy in patients with subclinical signs of cardiotoxicity [70]. Another study in breast cancer patients demonstrated a significant benefit of CRF and quality of life after a 16-week high intensity exercise intervention (AET or RT) during chemotherapy compared to UC [71]. These results were confirmed in a multi-center trial in early-stage BC patients on AC-based chemotherapy showing an increase of the secondary endpoint VO_2peak_ at the end of chemotherapy (+ 1.6 ml/kg/min, *p* = 0.041) and after 3 months (+ 3.1 ml/kg/min, *p* < 0.001) with combined AET and RT compared to UC [50•]. However, there was no difference for the primary endpoint change in LVEF at the end of chemotherapy (0.7%, *p* = 0.349) and 3-month follow-up (1.1, *p* = 0.196), or the secondary endpoint GLS (− 0.3, *p* = 0.500 and − 1.0, *p* = 095), which may either suggest that follow-up was too short, or that functional rather than echocardiographic variables may better represent early onset cardiotoxicity; this clearly needs further investigations.

The Brexit trial, the cancer exercise study with the longest follow-up period so far, demonstrated a lower percentage of “functional disability,” defined as VO_2peak_ ≤ 18 ml/kg/min (12 vs. 36%) after a 12 months follow-up in an exercise training group consisting of combined supervised and home-based AET (including MCT and HIIT elements) and RT, compared to UC. The training group displayed a 9% improvement of VO_2peak_ compared to a 7% decline in UC [65•]. Exercise did not influence resting LVEF, but improved left and right ventricular ejection fraction reserve and stroke volume and led to a lower increase of post-chemo troponin compared to UC. A tremendous and clinically relevant net increase of 3.5 ml/kg/min of VO_2peak_ in the AET group raises the question whether current definitions of CTRCD, which are based on resting and laboratory markers, should be enriched by functional exercise variables to better risk stratify cancer patients. This trial also suggests a combination of MCT and HIIT in a non-linear training model combining effects on VO_2peak_; clearly, this needs further investigation in a long-term follow-up.

Apart from improvement of CRF (VO_2peak_), AET was shown to mitigate cardiovascular risk factors, such as sarcopenic obesity [72] and inflammatory markers [73] in breast CS, cholesterol profile in prostate cancer [74, 75] and survivors of childhood cancer [76], as well as endothelial function in breast and prostate cancer survivors [77, 78]. Structured pre-operative AET also seems to reduce post-operative complications and length of hospital stay [79] and facilitates efficacy of cancer therapy [80].

Recurrence and mortality of cancer were reduced following a 6-month exercise trial after colon cancer surgery [81]. Although heterogeneity of study designs were reported, similar results were gained in a meta-analysis of eight randomized controlled exercise trials in cancer patients showing a reduced risk of cancer recurrence in exercising individuals and a lower risk of mortality [82].

In breast cancer patients exercise training has shown positive effects on CRF and reduction of cancer-related symptoms in patients before initiation of chemotherapy [45]. Likewise, exercise can modulate angiogenic factors and tumor biology during neoadjuvant therapy, which may have positive effects on treatment response [83]. Exercise training is safe during chemotherapy [48, 69] and can even improve CRF during this period [50•]. Several studies demonstrated positive effects of exercise on CRF, cardiovascular events, and survival after adjuvant chemotherapy [65•, 68, 84, 85]. Exercise training also demonstrated reduction of arthralgia induced by aromatase inhibitors during maintenance therapy [86]. Professional support in cancer rehabilitation is of pivotal importance as structured and personalized exercise rehabilitation training in breast cancer survivors reduces fatigue and fatigue-related biomarkers [87], and can also improve social and physical function [88]. Taken together, evidence for the beneficial effects of exercise on patients’ well-being and CRF have been demonstrated across the whole temporal continuum of cancer treatment and should induce integration of exercise as a main pillar of standard-cancer therapy [89]. Future trials will also provide more insights on the effects of structured, individualized exercise to improve quality of life and fatigue in more advanced cancer stages, such as metastatic breast cancer [90].

Muscle wasting and sarcopenia is a major problem in cancer patients and contributes to a reduction of CRF and survival [91–93]. Resistance training (RT), as a measure to reduce or even reverse muscle wasting, is recommended in addition to endurance training to improve CRF by current guidelines of sports cardiology and cardio-oncology [8, 94] as it improves skeletal muscle and mitochondrial respiration [95]. Assessment of muscle strength in cancer patients is usually performed by measuring handgrip strength [96], and RT intensity to establish training prescriptions should be identified by the one repetition maximum (1 RPM) [89, 94]. A meta-analysis of randomized trials of RT during and after chemotherapy in cancer survivors demonstrated the most effective increase of muscle strength at intensities below 75% of 1 RPM [97] and provides evidence for a safe integration of RT into training of cancer patients.

Recent trials used combined AET and RT to demonstrate improvement of CRF in patients during and after chemotherapy [50•, 65•, 98], but did not include frail and sarcopenic patients [96, 99]. Future exercise studies in cancer patients need to take this into account and should also enroll sarcopenic cancer patients who should be trained with combined RT and AET and additional structured nutritional advice. Trials are warranted to gain data on which RT intensity before, during, and after chemotherapy is most suitable to improve muscular strength and antagonize sarcopenia and muscle wasting. Following principles of exercise sciences, variations of exercise intensity, duration, and frequency should be applied in cancer patients. Repetitive re-assessments of training corridors throughout the treatment periods should be implemented for both AET and RT (a prospective design for exercise intervention has been suggested by our group, [89]). As exercise interventions in trials of sports cardio-oncology are heterogeneous, exercise prescriptions should be based on objective measures gained by repetitive cardiopulmonary exercise testing and handgrip strength assessment. Repetitive testing is of pivotal importance, since drug therapy may change throughout the treatment cycles, which may affect cardiopulmonary response to exercise and alter pre-defined training corridors (e.g., the implementation of beta-blockers to treat left ventricular dysfunction and heart failure). Analogous to cardiac sports groups, cancer patients should primarily receive supervised training in the form of “cancer sports groups,” which should be enacted by trained personnel. These training sessions may be performed in specialized facilities, or even within hospitals, depending on the patients` health status and extent of functional disability. Prescriptions of exercise should be done by professional exercise scientists in close cooperation with the treating physicians. Supervised training should be preferred as it may be more effective and safer compared to home-based training. However, in less compromised patients, the advent of telemonitoring and app-based solutions may bear the potential to improve home-based training with maintenance of adequate exercise monitoring. Future trials of sports cardio-oncology should make use of this digital progress [89].

## Variation of Exercise Intensity: HIIT Vs. MCT

Variations of exercise intensity are established approaches to increase exercise performance, but data on the net benefit of MCT versus HIIT is conflicting. In healthy individuals a meta-analysis of 28 trials demonstrated a greater improvement of VO_2peak_ in HIIT compared to MCT [100]. HIIT was introduced in the form of the “Norwegian model” into cardiological trials by comparing post-infarct HFrEF patients under optimal medical therapy into an MCT (70% of peak heart rate), HIIT (95% of peak heart rate, 4 × 4 min, 3x/week for 12 weeks), or UC group [101]. HIIT outperformed MCT in changes of VO_2peak_, LV diameters, LVEF, NTproBNP, and surrogate markers for mitochondrial and endothelial function [101]. These results were not confirmed in a larger randomized trial of patients with LVEF ≤ 35%, in which almost half of the patients did not adhere to the recommended training corridors [102]. Although a cohort of patients with HF and HFpEF displayed no differences in VO_2peak_ improvement between MCT and HIIT [103], a meta-analysis of three RCT HFpEF trials recently demonstrated a benefit for HIIT [104]. When comparing isocaloric HFrEF studies, MCT and HIIT did not differ in VO_2peak_ [105] or LVEF improvement at rest [106].

A meta-analysis of 12 studies with cancer patients and survivors compared CPET data after exercise intervention and found no differences of VO_2peak_ between MCT and HIIT, both being superior to UC [107]. However, heterogeneity of included trials and lack of objective pre-intervention CPET data limit this study`s generalizability. Compared to UC, HIIT proved to be superior to improve VO_2peak_ and cardiovascular risk factors, such as LDL-cholesterol or arterial stiffness, in different types of cancer [108, 109]. HIIT was also shown to modulate sympathetic tone [110], which may be a driving mechanism to develop HF in cancer. Apart from VO_2peak_ improvement, positive effects of HIIT on quality of life have been described in cancer patients and CS [71, 111], but more data needs to be acquired on long-term safety and whether HIIT, as a time-sparing alternative or complementary approach, is better suited than MCT to facilitate chemotherapeutic response through decrease of tissue hypoxia [112].

Although pre-clinical and clinical trials on exercise have been published to mitigate cardiotoxic effects of cancer therapy, improve CRF, and reduce overall mortality [113], the protective molecular mechanisms of exercise leading to peripheral and central adaptations have not been entirely elucidated. Extracardiac contributions to the increase of VO_2peak_ depending on specific exercise regimens have not been sufficiently elucidated in exercise trials of cancer patients. Deeper knowledge of molecular processes of different exercise regimens in cancer patients would facilitate designing more targeted training strategies.

## Molecular Biology of Exercise Regimens

**Fig. 1:**
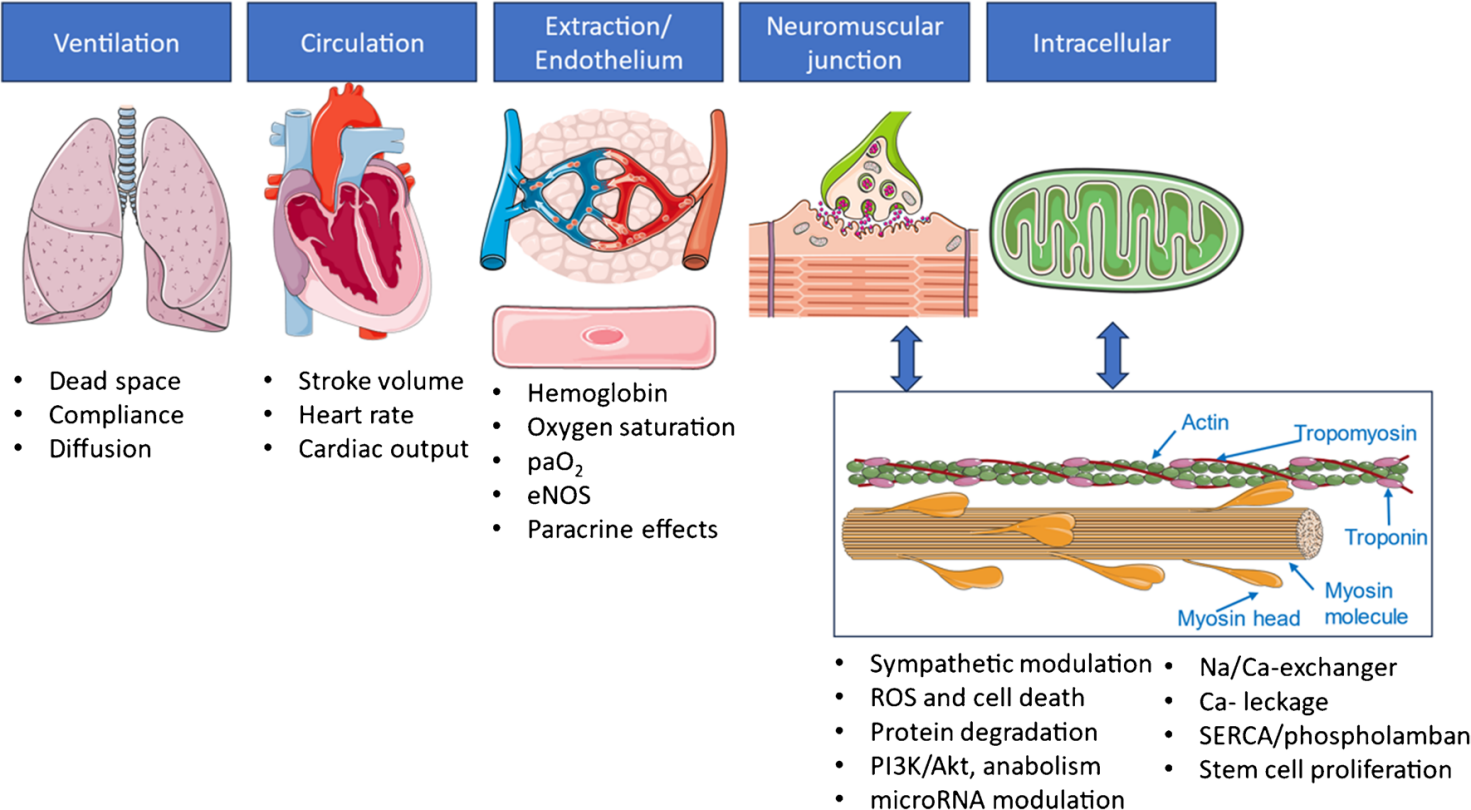
Impact of exercise training on macroscopic and microscopic structures and processes. Oxygen, which is transported along the upper and lower respiratory tract, diffuses into the vascular system and is delivered to the peripheral working musculature, where it is extracted from cells. Determinants of cardiac output, the driver of circulation, are stroke volume and heart rate increase during exercise. The amount of intracelluar oxygen uptake depends on arterial oxygen content, which is a function of partial pressure of oxygen (p_a_O_2_), oxygen saturation and hemoglobin levels, as well as endothelial function, which is, among others, regulated by endothelial nitric oxide-synthase (eNOS) and crosstalk with microRNA. Paracrine mediators between muscle cells (myo- and mitokines) and nerval modulation at the neuromuscular junction modulate intracellular downstream signaling. A balance between reactive oxygen species (ROS), programmed cell death and protein degradation (ubiquitination) as well as hypertrophy mediated through the phosphoinositid-3-kinase/Akt cascade (PI3K/Akt) is achieved through structured exercise training leading to physiological myocardial remodeling in the form of the athletic heart. Modulation of calcium sensitivity through regulation of membrane-bound Na^+^/Ca^2+^-exchanger, sarcoplasmic/endoplasmic reticulum Ca^2+^- ATPase (SERCA) and phospholamban is another mechanism to optimize myocardial actin-myosin interaction. Upregulation of mitochondrial and respiratory chain enzymes lead to more effective aerobic energy disposal. Partial renewal of myocardial cells via stem cell proliferation can also be triggered through increased workload. Created with Biorender.com

Exercise has been suggested as cardiovascular therapy more than 2 decades ago [114] and knowledge on macroscopic and microscopic adaptations to training has increased since then, with beneficial effects on organ, vascular, and cellular structures and processes (for an overview see Fig. 1).

A major problem of cancer is cardiac and muscle wasting [115]. Balance between cell growth (also as a driver of cancerogenesis) and atrophy is maintained through the ubiquitin–proteasome system (UPS) [116] and myostatin-signaling [117], as well as auto- and mitophagy [118–120]. MCT has been shown to counteract muscle wasting in a heart failure animal model [121] and in humans [122] by reducing muscle ring finger 1 (Mu-RF1) expression, which is involved in skeletal muscle wasting. After 12 weeks of MCT, a significant reduction in myostatin expression in 24 HF patients was observed compared to UC [123]. MCT has also been shown to stimulate IGF-1, which triggers phosphoinositid-3-kinase (PI3K) and Akt expression leading to (physiological) myocardial hypertrophy [124], while this pathway may be inhibited through exercise in certain cancer cells [125, 126].

Reactive oxygen species (ROS), which can be triggered by increased expression of NADPH and xanthine oxidase leading to myofibrillar damage [127], is another mechanism to drive exercise intolerance: MCT proved to decrease the amount of nitrotyrosine and carbonylated proteins, thus reducing ROS load on muscular cells [128]. MCT can also induce modification of microRNA, which is involved in cellular morphogenesis, antifibrotic metabolism, inflammation, and cell death, which has been identified to preserve cell integrity [129]. Facilitation of paracrine signaling [130], NO-synthase [131], and neuromuscular transmission [132], as well as an increase of mitochondrial density [133], can be achieved through MCT. AET (MCT and HIIT) also leads to increased activity of mitochondrial and respiratory chain enzymes through activation of peroxisome proliferator-activated receptor-gamma coactivator-1 protein-alpha (PGC-1α) and may be downregulated by cancer cells [80, 115, 118, 134–137]. There is conflicting data whether facilitation of endothelial NO synthase (eNOS), as a surrogate to increase endothelial function, is best achieved through HIIT or MCT [101, 138].

HF leads to reduced Ca^2+^- uptake into the sarcoplasmatic reticulum due to diminished activity of sarco/endoplasmatic reticulum Ca^2+^ -ATPase (SERCA) [139], which may also be one mechanism of cancer-related exercise intolerance [118]. HIIT has been shown to increase expression of Na/Ca^2+^ exchanger [140] and triggers hyperphosphorylation of phospholamban [141] leading to improved contractility of cardiomyocytes. Formation of new, functional cells through exercise may also be a mechanism, which could enhance exercise tolerance in cancer patients: Wistar rats exercising with MCT (55–60% of VO_2peak_) and HIIT (85–90% of VO_2peak_) for 30 min/day, 4 days/week, and for 4 weeks both displayed significantly increased newly formed cardiomyocytes compared to sedentary controls, with the highest number gained in HIIT [142].

In summary, both HIIT and MCT interact with multiple pathways leading to improved central and peripheral adaptations to exercise. Taking advantage of such exercise-induced effects without fostering cancer growth will be one major task of future trials of sports cardio-oncology.

## Exercise Volume as a Function of Intensity, Frequency, and Duration: Significance for Planning Future Exercise Trials in the Cancer Population

Like in healthy individuals, a minimal amount of exercise volume must be applied in cancer patients to improve CRF [8, 94, 143]. Current clinical exercise trials have focused on examining variations of exercise intensity (HIIT vs. MCT) with relatively constant exercise duration (30–45 min) and frequency (3 × /week) [144, 145]; the impact of the latter two on exercise volume, which is the product of intensity, duration, and frequency, remains to be determined. Scarce data in humans is available for mitochondrial adaptations to exercise by increasing frequency and duration [144]. The most appropriate exercise regimen to improve capillary density (and reduce potential tumor-associated hypoxia in cancer patients) in skeletal muscles of humans has not been determined [144].

An exercise regimen, which has not been investigated in the cancer population, is low-volume sprint interval training (SIT), which requires less total work to complete relative to MCT and HIIT and is performed at intensities close to VO_2peak_ [146]. Similar to HIIT, SIT is better able to upregulate mitochondrial density (mediated through AMPK and p38 MAPK phosphorylation and PGC-1α increase) [147] and content (measured by activity of citrate synthase) [148] after each training session relative to MCT.

Current evidence in healthy subjects suggests that improvement of VO_2peak_ is rather gained through preservation of intensity (with preferably longer bouts) than duration or frequency [149], but data is lacking in cancer patients. In healthy subjects, increase of VO_2peak_ has been primarily attributed to increases of stroke volume after up to 6 weeks of training rather than by enhancing arteriovenous O_2_ difference [150]. There is an urgent need to investigate additional mechanisms of central and peripheral adaptations in exercising cancer patients with overt or occult cardiotoxicity and co-existing HF medication to treat CTRCD. For clinical practice, the benefits and exercise-limitations of drugs limiting cardiac output (such as beta-blockers) have to be weighed carefully in cancer patients in the absence of clear indications, such as reduced LVEF, or a history of myocardial infarction. This should be regarded similarly to treating “normal” HFpEF patients, for whom beta-blockers have been shown to display severe exercise limitations [10, 151, 152].

In summary, an array of molecular adaptations have been demonstrated in different exercise regimens in healthy, cancer, and HF patients [145, 153], some of which are antagonized by cancer cells [83]. The diverse molecular response to exercise regimens suggests that AET (MCT, HIIT, and low-volume SIT) exerts its effects on multiple complementary pathways, which differ from established single-pathway inhibition of standard cancer therapy and may qualify exercise as a “pleiotropic drug” in cardio-oncological therapy. However, finding the most appropriate training regimen to reduce the risk for cardiotoxicity and CTRCD and eventually prognosis of cancer patients will require both further clinical and molecular studies in exercise sciences. It should also be critically discussed whether the definition of CTRCD, which entirely depends on resting measurements, should be enriched by functional (at best CPET) data to better risk stratify cancer patients. Studies should not only be performed to investigate hard clinical endpoints but also should be designed to include analysis of exercise-induced molecular changes on and off chemotherapy as well as during different cycles of exercise training to better understand the pathophysiology of CTRCD and beneficial effects of exercise. As cancer and HF-induced catabolism is a major concern, training should always integrate RT and nutritional protocols to foster protein synthesis accordingly. Importantly, exercise interventions, including MCT, HIIT, and SIT must follow principles of exercise sciences using CPET as a gold standard for testing, instead of estimating exercise intensity from predicted peak heart rate. By doing this, heterogeneity of trials in sports cardio-oncology will be reduced.

## Conclusion

Physical inactivity is not only a risk factor to develop cancer but is also a predictor for HF and cardiovascular death [154]. Exercise interventions, both AET (MCT and HIIT) and RT, during chemotherapy have been shown to be safe and applicable [155, 156] and have the potential to improve CRF and prevent development of long-term HF [97, 157, 158] by affecting multiple molecular pathways. This should trigger the implementation of exercise training as an adjunct to standard cancer-related therapy in the form of cardio-oncological rehabilitation, which is not generally available despite “guideline recommendations” [8, 159–162]. More exercise trials are needed to elucidate the most appropriate training regimen to prevent and antagonize CTRCD, whose definition may require revision in the future to incorporate additional functional variables.

## Data Availability

We did not perform analysis of original data. All information is included in this manuscript.
